# Polypyrrole–Tungsten Oxide Nanocomposite Fabrication through Laser-Based Techniques for an Ammonia Sensor: Achieving Room Temperature Operation

**DOI:** 10.3390/polym16010079

**Published:** 2023-12-26

**Authors:** Mihaela Filipescu, Stefan Dobrescu, Adrian Ionut Bercea, Anca Florina Bonciu, Valentina Marascu, Simona Brajnicov, Alexandra Palla-Papavlu

**Affiliations:** National Institute for Laser, Plasma and Radiation Physics, RO-077125 Magurele, Romania; mihaela.filipescu@inflpr.ro (M.F.); stefan.dobrescu@inflpr.ro (S.D.); bercea.adrian@inflpr.ro (A.I.B.); anca.bonciu@inflpr.ro (A.F.B.); valentina.marascu@inflpr.ro (V.M.); brajnicov.simona@inflpr.ro (S.B.)

**Keywords:** polypyrrole, tungsten oxide, ammonia, gas sensor, laser, pulsed-laser deposition, matrix-assisted pulsed-laser evaporation

## Abstract

A highly sensitive ammonia-gas sensor based on a tungsten trioxide and polypyrrole (WO_3_/PPy) nanocomposite synthesized using pulsed-laser deposition (PLD) and matrix-assisted pulsed-laser evaporation (MAPLE) is presented in this study. The WO_3_/PPy nanocomposite is prepared through a layer-by-layer alternate deposition of the PPy thin layer on the WO_3_ mesoporous layer. Extensive characterization using X-ray diffraction, FTIR and Raman spectroscopy, scanning electron microscopy, atomic force microscopy, and water contact angle are carried out on the as-prepared layers. The gas-sensing properties of the WO_3_/PPy nanocomposite layers are systematically investigated upon exposure to ammonia gas. The results demonstrate that the WO_3_/PPy nanocomposite sensor exhibits a lower detection limit, higher response, faster response/recovery time, and exceptional repeatability compared to the pure PPy and WO_3_ counterparts. The significant improvement in gas-sensing properties observed in the WO_3_/PPy nanocomposite layer can be attributed to the distinctive interactions occurring at the p–n heterojunction established between the n-type WO_3_ and p-type PPy. Additionally, the enhanced surface area of the WO_3_/PPy nanocomposite, achieved through the PLD and MAPLE synthesis techniques, contributes to its exceptional gas-sensing performance.

## 1. Introduction

Nowadays, there is a growing interest in low-cost, high-performance environmental pollution monitoring devices, particularly sensors. Daily emissions of toxic gases from both natural and human-made sources pose significant environmental and health risks, contributing to climatic disturbances and respiratory ailments. Small amounts (parts per million (ppm)) of toxic gases can be detected with different types of sensors (resistive [1], capacitive [2], and surface acoustic wave [3]).

The active materials in most sensors on the market are based on metal oxide semiconductors, such as SnO_2_, ZnO, or WO_3_ [4]. Tungsten oxide in particular is recognized for its versatility in detecting both oxidizing and reducing gases, especially in ammonia sensing. Thus, numerous methods, i.e., hydrothermal synthesis of nanoflakes [5], microwave-assisted hydrothermal synthesis for obtaining nanoparticles [6], electrospinning for nanofibers [7], or pulsed-laser deposition (PLD) [8] have been employed to prepare WO_3_ with different shapes, resulting in sensitive gas sensors with notable responses to NH_3_, NO_2_, acetone, and ethanol.

However, the main issue with metal oxide semiconductor-based sensors is their high operating-temperature requirement (often in the range of 200 to 500 °C) to operate effectively. This elevated temperature is necessary to facilitate the chemical reactions involved in gas-sensing processes, such as the adsorption and desorption of gas molecules on the sensor surface. Therefore, efforts are being made to develop sensors that operate at lower temperatures without compromising sensitivity and selectivity. This involves exploring alternative materials, modifying surface properties, and employing advanced fabrication techniques to enhance the sensor’s performance at room temperature or moderately elevated temperatures.

Polymers (such as polyaniline (PANI), polypyrrole (PPy), polythiophene, and poly(3,4-ethylenedioxythiophene)) have been extensively explored as sensing materials owing to their advantages of low operating temperature, cost effectiveness, and flexibility. However, the drawback of these polymers lies in their inherent limitations of poor stability and long response time [9,10]. To address these challenges, there is great interest in the development of hybrid inorganic–organic composite materials as thin layers which can pose enhanced physical and chemical properties due to their complementary behaviours [11,12]. Polymer composite-based ammonia sensors operating at room temperature have been the subject of numerous research studies. For instance, sensing membranes employing PANI have been fabricated, for example, PANI–halloysite nanotubes [13], CeO_2_ NP@PANI hydrogel nanohybrids [14], PANI–carbon nanotubes [15], or PANI–MXene [16], exhibiting substantial response at various concentrations of NH_3_ at room temperature. Another polymer of interest for ammonia detection is polypyrrole due to its relatively high electrical conductivity among conducting polymers, environmental stability, and chemically inert behaviour. Also, PPy generally has good mechanical strength, which is advantageous in situations where the sensor may experience mechanical stress or deformation (flexible sensors) [17]. Sensitive layers of PPy in different configurations (thin films, nanofibres, nanowire array, and nanotubes) have been obtained by different methods (chemical polymerization, Langmuir–Blodgett, vapour-phase polymerization, electro-polymerization, etc.) and tested, showing good responses to ammonia different concentrations at room temperature [18].

Recently, WO_3_-PPy composites have gained attention due to their chemical stability, enhanced sensitivity and selectivity to toxic gas (NO_2_, H_2_S, acetone, triethylamine) [19,20,21,22,23], and lower operating temperatures. Compared to some other sensing materials, tungsten oxide and polypyrrole have the advantage of being cost effective. This can be important for the widespread deployment of sensors in various industrial or environmental monitoring situations.

In this work, we will demonstrate resistive gas sensors with nanocomposites based on conductive polymers and metal oxide semiconductors as active materials, with a specific focus on achieving optimal performance at room temperature. In particular, we focus on applying laser-based methods for the synthesis of nanocomposites of PPy and WO_3_. Although there are numerous publications which demonstrate the fabrication of gas sensors with PPy and WO_3_ nanocomposites [24], there are still many challenges to overcome, such as the low adherence of the materials to the substrate, tuning of the energy barrier at the electrode–active material interface for easy charge transport, etc. Thus, we use laser methods, i.e., pulsed-laser deposition (PLD) [25] and matrix-assisted pulsed-laser evaporation (MAPLE) [26] to process different types of materials, both organic and inorganic, into layers with controlled thickness, structure, and topography onto metallic electrodes.

Our work aims to contribute to the advancement of gas sensors by showcasing the efficacy of resistive gas sensors based on PPy and WO_3_ nanocomposites synthesized through PLD and MAPLE. Ultimately, this work provides a pathway towards reliable, room-temperature-operable gas-sensing devices with enhanced sensitivity.

## 2. Materials and Methods

### 2.1. Materials

Sensitive composite layers based on tungsten oxide (WO_3_) and polypyrrole (PPy) polymer are prepared both on Si (100) substrates from Neyco (Vanves, France) (for postcharacterization measurements, i.e., infrared spectroscopy) as well as on substrates prepatterned with metallic interdigital electrodes (DRP-G-IDEAU10 from Metrohm DropSens, Oviedo (Asturias) Spain), using PLD and MAPLE techniques.

A WO_3_ ceramic target (99.9% purity) (Kurt J. Lesker Company GmbH, Dresden, Germany) is employed for the pulsed-laser deposition (PLD) experiments. Solutions of polypyrrole polymer (Merck, Darmstadt, Germany) suspended in N-Methyl-2-pyrrolidone (NMP) (Honeywell Specialty Chemicals Seelze GmbH, Seelze, Germany) at a concentration of 1% are used for the preparation of the matrix-assisted pulsed-laser evaporation (MAPLE) targets.

### 2.2. Preparation of the Sensing Layers

**Figure 1:**
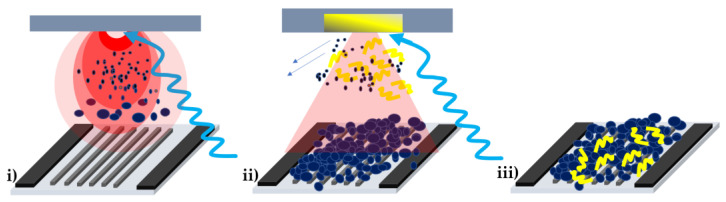
Sketch of the sensor fabrication: (**i**) mesoporous WO_3_ by PLD on interdigital electrodes; (**ii**) deposition of PPy layer by MAPLE on top of the WO_3_ mesoporous layer. The arrows indicate that the solvent molecules are pumped away from the deposition chamber, while the polymer material is deposited onto the WO_3_ layer; (**iii**) a WO_3_/PPy nanocomposite layer deposited onto the interdigital electrodes of a gas sensor.

A schematic illustration showing the preparation of the PPy/WO_3_ nanocomposite by PLD and MAPLE is depicted in Figure 1. In addition, single layers of WO_3_ and PPy are applied onto metallic electrodes by PLD and MAPLE, respectively. The active layer is applied on top of the interdigitated electrode area, using a mask to protect the two thick electrodes (1 mm) used for electric connections.

PLD is used for obtaining mesoporous WO_3_ layers onto which a thin layer of PPy is applied via MAPLE. Additional details about the setups and the method can be found in [8,27]. Briefly, the PLD experimental setup consists of a YAG:Nd pulsed laser (Quantel, France) (266 nm wavelength, repetition rate of 10 Hz, 7 ns pulse duration) and an in-house designed reactive chamber connected to vacuum pumps. The distance between the target and the substrate is fixed at 1 cm, and the laser fluence is set at 4.5 J/cm^2^. The WO_3_ target is ablated with 40,000 laser pulses. During WO_3_-layer deposition, in order to avoid any damage, the target is rotated, and the laser beam scans its surface with the help of an opto-mechanical system. The experiments take place at atmospheric pressure (1000 mbar air), a pressure that leads to a mesoporous architecture of the WO_3_ layer.

The MAPLE experimental system consists of a YAG:Nd pulsed laser (Continuum, Corbas, France) (266 nm wavelength, repetition rate of 10 Hz, 5–7 ns pulse duration) and a commercially available MAPLE reactive chamber from Neocera (Beltsville, MD, USA). To keep the target frozen during deposition, a system containing copper pipes connected to the target holder allows a continuous flow of liquid nitrogen from an external vessel. All MAPLE experiments are carried out in a vacuum (working pressure of 5 × 10^−^^5^ mbar). The distance from the target to the substrate is set at 3.8 cm. The laser fluence is 0.258 J/cm^2^.

### 2.3. X-ray Diffraction (XRD) Characterization

The crystallographic characteristics of the sensitive layers and target materials are investigated by X-ray diffraction (XRD). The XRD spectra are acquired with a PANalytical X’Pert MPD diffractometer with Cu-kα radiation in Bragg–Brentano θ-2theta geometry. The XRD measurements are carried out in the 10–60 2theta interval, with a step size of 0.02 and an acquisition time of 10 s/step. Data processing is performed using HighScore Plus software 5.1 and PDF-4+ Database.

### 2.4. Infrared (IR) Spectra

Chemical investigations of the as-deposited thin films are carried out by using the Fourier transform infrared spectrometer (FTIR) Jasco (Tokio, Japan) 6300 Type-A in transmission mode, in the 500–4000 cm^−1^ range, and at a resolution of 4 cm^−1^. The obtained spectra are processed by using Spectra Manager v.2 package software. For this measurement type, the thin films are deposited on FTIR-dedicated thin polished Si substrates (Neyco Company Supplier, Vanves, France). Silica gel is placed inside the spectrometer in order to reduce the atmospheric water vapours during FTIR measurements.

### 2.5. Raman Spectra

Raman spectroscopy is carried out using a DXR Raman Microscope (Thermo Fischer Scientific, Waltham, MA, USA) with a laser line of 532 nm as the excitation source. The spectra are obtained following 10 scans, with 20 s exposure time. The Raman spectra are collected over the 200–2000 cm^−1^ range at room temperature.

### 2.6. Water Contact-Angle Measurements

Water contact-angle measurements are carried out employing the sessile drop method with a KSV CAM101 microscope (KSV Instruments Ltd., Helsinki, Finland) equipped with a video camera. The contact-angle measurements are conducted by dispersing water droplets with a volume of 2.5 ± 0.5 µL. For each sample, contact-angle measurements are taken at five distinct points, and the reported contact angle is the average of these measurements.

### 2.7. SEM and AFM Measurements

We explore the surface morphology of the samples by scanning electron microscopy (SEM). Our analyses are carried out using the Scios 2 DualBeam, an advanced ultra-high-resolution analytical focused ion beam scanning electron microscope FIB-SEM system (Thermo Fisher Scientific Inc., Hillsboro, OR, USA), at voltages up to 30 kV.

The AFM images are acquired using an XE100 microscope (Park System, Suwon, Republic of Korea). The scanning of the surfaces was carried out with an OMLC-AC160-TS tip from Olympus (Tokyo, Japan), in noncontact mode at ambient conditions. Multiple regions and dimensions (40 × 40 µm^2^ and 5 × 5 µm^2^) are investigated to obtain data on the surface root-mean-square roughness (Rq) of the as-deposited layers.

### 2.8. Gas-Sensing Measurements

The ammonia-gas-sensing properties of the PPY and WO_3_/PPy sensors are investigated at room temperature (22 ± 1 °C), while the sensing properties of WO_3_ are evaluated at 100 ± 1 °C. The laser-fabricated sensors are inserted into a custom-built testing chamber containing a gas inlet and outlet. The electrodes of the sensor devices are connected to a Keithley 2450 SourceMeter (Keithley Instruments, Cleveland, OH, USA) which is operated in continuous mode. Two mass-flow controllers (MFCs, Alicat Scientific (Tucson, AZ, USA)) are used to deliver a mixture of various concentrations of NH_3_ in a carrier gas (N_2_) to the testing chamber with a total flow rate of 1 L/min (Figure S1). Various concentrations of ammonia gas in a range of 10–50 ppm are tested. An Alicat Scientific MC-10SLPM-D/5M MFC is used to control the N_2_ carrier gas flow rate, while an Alicat Scientific MC-10SCCM-D/5M MFC is used for controlling the NH_3_ flow rates. Flow rates are remotely controlled by connecting the MFCs to a laptop via a 6′ USB-MD8-232 double-ended 8-pin mini-DIN to USB serial cable (Alicat Scientific) and using Flow Vision 2.0 SC software (Alicat Scientific; available free of charge online) to change the flow rates. The humidity is measured using a hygrometer placed near the exhaust gas. Fifteen sensors (5 sensors for each of the PPy, WO_3_, and WO_3_/PPy sensing layers) were fabricated and tested. The response S is used to evaluate the sensor performance, i.e., S = (Rg − Ra)/Ra × 100% [28]. Here, Ra and Rg are the resistance of the sensor under dry nitrogen gas and the given concentration of ammonia, respectively. The response/recovery time is calculated as the time taken for the sensor to reach 90% of the total resistance change from its initial resistance.

## 3. Results and Discussion

### 3.1. XRD Spectra of WO_3_, PPY, and WO_3_/PPY Sensing Layers

**Figure 2:**
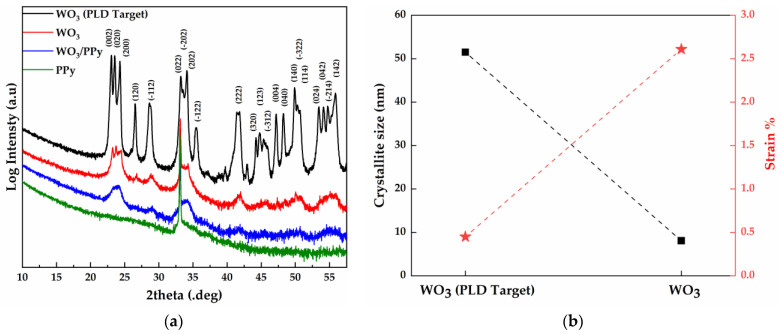
X-ray diffraction result of the active layers (**a**) XRD pattern of the WO_3_ target, WO_3_ thin layer, PPy thin layer, and WO_3_/PPy nanocomposite layers deposited by PLD and MAPLE; (**b**) crystallite size and strain of the WO_3_ target material and WO_3_ particles obtained by PLD. The data are obtained from the Sherrer Equation in HighScore Plus software.

The X-ray diffraction patterns of the active layers, together with the WO_3_ target and the PPy powder, are presented in Figure 2a. The presence of multiple diffraction peaks in the WO_3_ target spectra indicates the polycrystalline nature of the material, characteristic of the crystal structure of monoclinic WO_3_ (JCPDS file:043-1035 [29]). The WO_3_ layer obtained by PLD (red pattern in Figure 2a) shows similar characteristics to the original WO_3_ (PLD Target). No diffraction peaks are visible in the PPy powder spectra, and the narrow peak observed at 2theta = 33.100° is a reflection of the silicon substrate [30]. The addition of PPy on top of WO_3_ (WO_3_/PPy, Figure 2a) by MAPLE makes the (002), (020), and (200) individual lattice planes not easily distinguishable, but the main aspect of the diffraction pattern is maintained. The presence of PPy may reduce the visibility or intensity of diffraction peaks by preventing the X-rays from interacting effectively with the underlying WO_3_ [31].

The crystallite size and strain of the WO_3_ particles from both the WO_3_–PPy nanocomposite layer and the WO_3_ PLD target are presented in Figure 2b. The data plotted for both crystallite size and strain represents average values calculated from all visible diffraction peaks. However, for the WO_3_–PPy sensitive layer, the values of crystallite size and strain could not be extracted due to the limited distinguishability between the (002), (020), and (200) individual lattice planes. Nevertheless, it is reasonable to assume that the addition of PPy by MAPLE (a low-energy process) did not alter the crystalline characteristics of the WO_3_ particles made by PLD. Consequently, the values of crystallite size and strain obtained for WO_3_ are considered representative of the WO_3_/PPy sensitive layer as well.

The WO_3_ PLD target has a characteristic crystallite size of approximately 50 nm and a value of 0.45% strain. After PLD deposition, the WO_3_ particles have an average crystallite size of ~8 nm. This decrease in crystallite size is linked to the mechanism of WO_3_ particle generation by PLD. When the plasma plume is rapidly cooled at high pressure (1 bar of synthetic air) the vaporized atoms and molecules undergo nucleation, a process where these species collide, combine, and form clusters or nuclei that further grow into particles (with smaller crystallite size) through coalescence and aggregation processes [32,33]. Moreover, the reduction in crystallite size is accompanied by a notable increase in strain from 0.45% (PLD Target) to ~2.6% for the WO_3_ particles. This trend has been reported previously in [34], where ZnO, TiO_2_, SnO_2_, and MoO_3_ highly porous layers were fabricated by PLD in air, at atmospheric pressure, in an experiment similar to ours.

Moreover, according to previous studies, the shape and size of metal oxide nanocrystallites are of particular interest, as they provide energetically distinct adsorption sites for analytes on different crystal facets. Notably, our XRD studies reveal a nanocrystallite size of around 8 nm, aligning with the literature findings that suggest such dimensions are favourable for improving sensor performance, i.e., for WO_3_ nanoparticles with diameters of 25 nm; the sensitivity values towards 10 ppm NO_2_ and 200 ppm NO at 573 K are three to four times as large as those for D > 33 nm [35].

### 3.2. IR and Raman Spectra of WO_3_/PPy Active Layers in Resistive Gas Sensors

**Figure 3:**
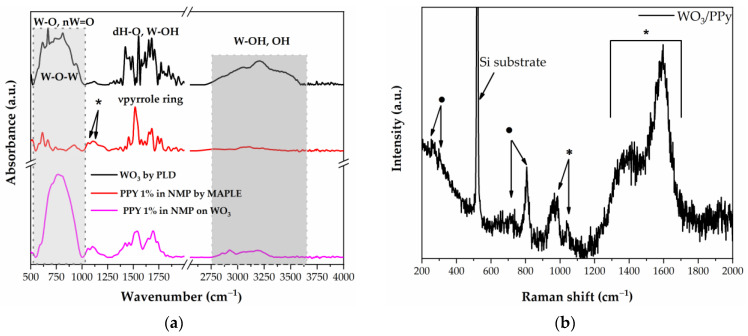
(**a**) FTIR spectra of WO_3_, PPy, and WO_3_/PPy nanocomposite as deposited by PLD and MAPLE. (**b**) Raman spectra of WO_3_/PPy nanocomposite. In the Raman spectra, the peaks marked with the symbol (*) are assigned to PPy polymer, whilst the peaks marked with (·) are assigned to WO_3_.

The as-deposited layers are measured via FTIR and Raman spectroscopy in order to investigate possible chemical changes that might occur due to laser processing. The FTIR bands visible in the WO_3_/PPy spectra shown in Figure 3a are in good agreement with those reported in the literature for polypyrrole and tungsten oxide [36,37]. The bands between 550–580 cm^−1^ occur due to W-O vibration, followed by the vibrations of νW=O around 950 cm^−1^, and W-O-W around 810 cm^−1^. In addition, the band at 1196 cm^−1^ is due to the C–N stretching vibrations, while those at 1294 cm^−1^ and 1049 cm^−1^ are due to the = C–H in-plane vibrations. Furthermore, the bands at 1554 cm^−1^ and 1474 cm^−1^ are attributed to the fundamental vibrations of the polypyrrole ring. The FTIR results exhibit a successful combination of both PPy and WO_3_.

The Raman spectra of PPy/WO_3_ are shown in Figure 3b. The spectra of the nanocomposite material show the characteristic peaks of both PPy (see also Figure S2) and WO_3_ (see also Figure S2), which indicates that no chemical damage occurs as a result of laser processing. In the spectrum of PPy, the peaks at 1552 cm^−1^ and 1340 cm^−1^ arise from the π-conjugated structure and ring stretching mode of the polymer backbone, respectively. The peak at 1037 cm^−1^ can be ascribed to the C–H in-plane deformation, and the two peaks at 925 cm^−1^ and 960 cm^−1^ correspond to ring deformation of the quinoid polaronic and bipolaronic structure, respectively [38]. Moreover, the ν(O–W–O) stretching mode of m-WO_3_ (at 807 cm^−1^ and 717 cm^−1^) and the δ(O–W) bending mode (273 cm^−1^ and 326 cm^−1^) can be attributed to a monoclinic γ-WO_3_ phase and are in good agreement with the XRD analysis [39]. These results are also consistent with the FTIR results.

### 3.3. Water-Contact-Angle Measurements of WO_3_, PPY, and WO_3_/PPy Sensing Layers

**Figure 4:**
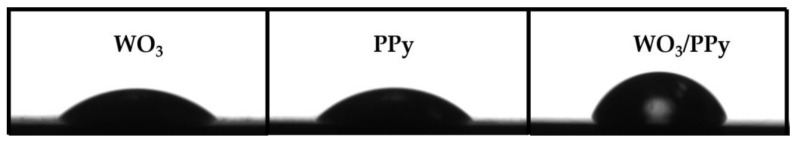
Contact-angle images for WO_3_, PPy, and WO_3_PPy sensing layers. The corresponding contact-angle average values for WO_3_, PPy, and WO_3_/PPy are 43.15°, 47.45°, and 64.92° respectively.

Contact-angle (CA) measurements are widely used for the assessment of the surface’s wettability, and the CAs measured on the different surfaces, i.e., WO_3_ by PLD, PPy by MAPLE, and the WO_3_/PPy nanocomposite are shown in Figure 4. The measured values are reported in Table S1. In designing a gas sensor, the choice of surface wettability, together with the active surface morphology and chemistry, as well as the overall sensor configuration play critical roles in determining the sensor’s performance.

Both the WO_3_ and PPy surfaces, as deposited by PLD and MAPLE, are hydrophilic, with CA of 43.15° and 47.45°. The surface of the WO_3_/PPy nanocomposite presents an increased CA, of around 65°, which shows that the surface is moderately hydrophilic. The increase in CA for the WO_3_/PPy layer can be elucidated by the distinctive topological features of the surface. The formation of mesopores results in a hydrophilic surface, and, in turn, the PPy layer fills the voids and intricacies of the WO_3_’s “fluffy” structure, imparting a moderately hydrophilic character to the interfaces. Our further studies will focus on designing hydrophobic and/or superhydrophobic surfaces and studying the response of the WO_3_/PPy nanocomposite towards ammonia. This is in particular advantageous, as ammonia is a polar molecule, and it tends to interact more favourably with hydrophobic surfaces due to the absence of water molecules on the surface. Additionally, hydrophobic surfaces repel water molecules which has the advantage of reducing interference from ambient humidity.

### 3.4. SEM and AFM Measurements of WO_3_, PPY, and WO_3_/PPy Sensing Layers

**Figure 5:**
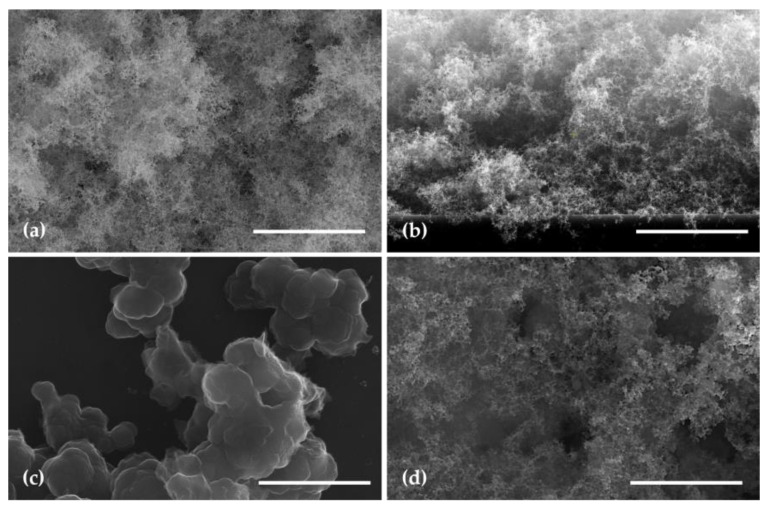
SEM images of a WO_3_ layer (**a**) top view; (**b**) cross section; (**c**) top view of a PPy layer; (**d**) top view of a WO_3_/PPy layer. The scale bar in all images is 3 µm.

The SEM images of Ppy, WO_3,_ and WO_3_/PPy nanocomposites are shown in Figure 5. As can be seen from the SEM images, the layers present different micro–nano structures depending on the experimental processing conditions. In particular, the WO_3_ layer exhibits a uniform and continuous morphology (as shown in Figure 5a). The film presents a distinctive mesoporous appearance and texture, with numerous interconnected pores or voids, forming an intricate network (see the SEM in the cross section in Figure 5b). Throughout the surface, there are irregularities resembling “fluffy clouds” with varying densities and heights. The thickness of the WO_3_ layers as deposited by PLD is in the 8 µm range. The size of these mesopores may vary, and some regions might display larger, well-defined pores, while others may have a more diffuse and interconnected network of smaller pores. This enhanced porosity is expected to contribute to a more favourable response, attributed to increased diffusion rates and greater gas-sorption capabilities.

Furthermore, the PPy layer deposited by MAPLE (20 nm thickness) reveals a textured surface with a granular appearance (Figure 5c). The granules, which make up the thin layer, vary in size and are densely packed, creating a three-dimensional topography. The granular structure of the PPy thin layer is indicative of the polymer’s inherently semicrystalline nature [40].

The SEM image shown in Figure 5d, i.e., the surface of the WO_3_/PPy nanocomposite layer, where PPy is applied on top of the mesoporous WO_3_ surface, exhibits significant porosity. The SEM image reveals that the PPy layer fills in the voids and intricacies of the WO_3_ “fluffy” structure. In areas where the tungsten oxide layer is denser, the PPy layer creates localized variations in the composite structure. The polymer layer may also penetrate into the pores of the tungsten oxide, creating a composite structure with enhanced interfacial interactions. The nanocomposite surface, as depicted in the SEM image, showcases the synergistic combination of the fluffy tungsten oxide layer and the PPy coating.

**Figure 6:**
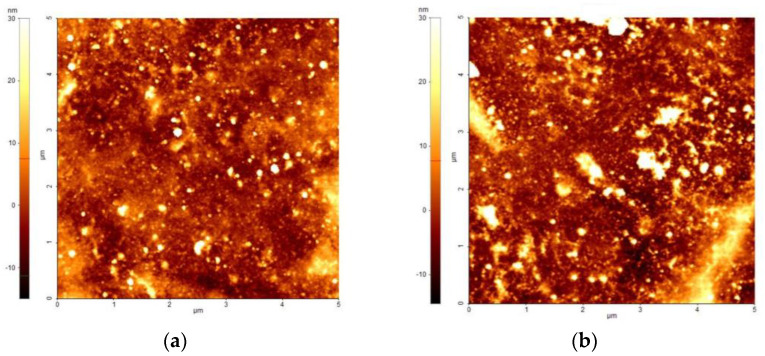
AFM images of the (**a**) PPy layer as deposited by MAPLE; (**b**) the WO_3_/PPy nanocomposite layer.

In addition, the AFM images shown in Figure 6 depict the surfaces of the PPy layer (Figure 6a), and of the WO_3_/PPy nanocomposite, respectively (Figure 6b). Due to the mesoporous structure of the WO_3_ layer as deposited by PLD, it was not possible to obtain reliable measurements. The WO_3_/PPy nanocomposite surface presents a heterogeneous topography, capturing the features of both the mesoporous tungsten oxide layer and the PPy coating (Figure 6b).

### 3.5. Gas Measurements of WO_3_, PPY, and WO_3_/PPy Sensing Layers

Tungsten oxide (WO_3_) is an n-type semiconductor with a broad band-gap energy (2.6−3.25 eV) known to possess excellent sensitivity towards a large number of gases at high temperatures [8]. In contrast, PPy is a polymer which exhibits p-type semiconductor characteristics, demonstrating a positive charge-carrier nature and the ability to accept electrons, making it an essential material in electronic and sensor applications. It is well-known that the synergistic effect of two sensing materials in nanocomposite always supports improved gas-sensing characteristics. Therefore, given the surface chemistry, structure, morphology, and topography of the WO_3_ sensing layers (i.e., mesoporous) combined with the PPy thin film (nanostructured), we evaluated the resistive response of the single and nanocomposite layers towards various concentrations of ammonia at temperatures close to room temperature (22 ± 1 °C).

**Figure 7:**
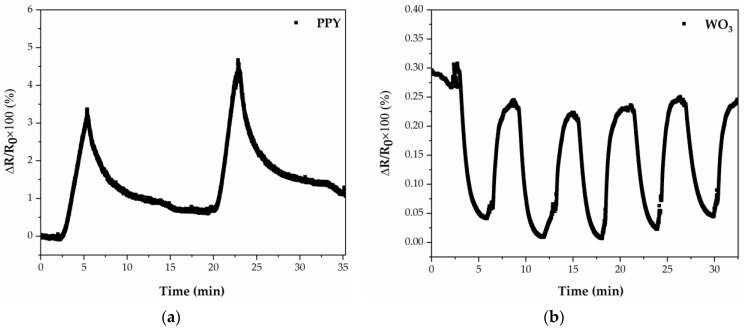
(**a**) Time-dependent response obtained at 22 ± 1 °C of a gas sensor with PPy active layer deposited by MAPLE; (**b**) Time-dependent response obtained at 100 ± 1 °C of a gas sensor with WO_3_ active layer deposited by PLD.

The PPy and WO_3_ sensor responses toward 50 ppm of ammonia are shown in Figure 7. In both cases, the sensors are exposed for 3 min to 50 ppm NH_3_, and then, dry N_2_ is introduced into the testing chamber. In the case of PPy active material (tested at 22 ± 1 °C), upon exposure to 50 ppm of NH_3_, the response of the sensor increases monotonically; when the ammonia-gas stream is stopped and nitrogen is introduced in the testing chamber, the sensor has a slower decrease upon removal of the NH_3_, of 15 min. The recovery of the sensor is not very fast, which might be partially related to the difficulty in the desorption of low NH_3_ concentrations. The PPy sensor shows an expected p-type semiconductor response upon exposure to a reducing gas (NH_3_), which is in accordance with previous reports [41,42].

In contrast to the PPy sensors fabricated by MAPLE, the sensors based on WO_3_ by PLD exhibit no room temperature response to NH_3_. Therefore, we have investigated their response at 100 ± 1 °C. In the case of WO_3_ active material, tested at 100 ± 1 °C, the sensor response decreases abruptly (the response time is 80 s) and then gradually approaches a steady state upon exposure to NH_3_. After purging with dry N_2_, the sensor recovers its baseline in 70 s. This is expected for a n-type semiconductor upon exposure to ammonia [8]. This is due to the fact that ammonia molecules tend to donate electrons to the WO_3_ surface, leading to an increase in the concentration of free electrons and, consequently, enhancing the electrical conductivity of the WO_3_ semiconductor. The repeatability of the WO_3_ sensor measured toward five exposure–recovery cycles of NH_3_/N_2_ gas under the same conditions at 100 ± 1 °C is also demonstrated in Figure 7b. The result shows that the WO_3_ sensor has good repeatability at 100 ± 1 °C.

**Figure 8:**
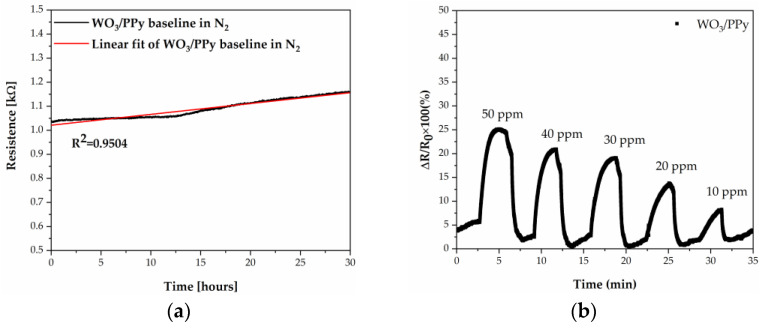
(**a**) Evolution of the WO_3_/PPy nanocomposite sensor baseline in time; (**b**) Time-dependent response obtained at 22 ± 1 °C of a gas sensor with a WO_3_/PPy active layer deposited by PLD/MAPLE.

Further on, we went on to explore the sensing properties of the WO_3_/PPy towards NH_3_. Prior to evaluating the WO_3_/PPy sensor response to ammonia, we evaluate the baseline resistance in time (i.e., for more than 30 h of continuous measurement), at 22 ± 1 °C, in a dry N_2_ atmosphere, and find that it is in the range 1–2 kΩ (see Figure 8a). Moreover, the baseline demonstrates an approximately linear trend, as indicated by the high R-square value of 0.95.

The NH_3_ sensing properties of the sensors based on the WO_3_/PPy nanocomposite toward different concentrations of NH_3_ (10–50 ppm of NH_3_) recorded at 22 ± 1 °C are shown in Figure 8b. The sensor response increases fast (the response time is 50 s, i.e., 40% faster than for WO_3_ at 100 °C), gradually approaches a steady state upon exposure to NH_3_, and recovers its initial baseline in 70 s (similar to the WO_3_ tested at 100 °C). The WO_3_/PPy nanocomposite sensors have an enhanced response to NH_3_ compared to its constituent materials, which we believe to be due to the synergistic effects, i.e., increased surface area, including film thickness and porosity, metal oxide–polypyrrole ratio, p–n heterojunction formation, and specific gas interactions inherent to the hybrid nanocomposite material. While factors such as film thickness and porosity contribute to sensitivity and response time [43,44], the interplay between metal oxide and polypyrrole in determining these characteristics requires further exploration in our future studies.

**Figure 9:**
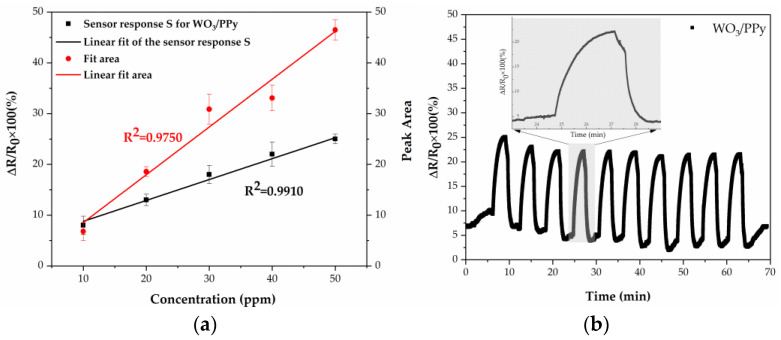
(**a**) Concentration-response and concentration-peak area relationships of the WO_3_/PPy nanocomposite layer. (**b**) Repeatability of the WO_3_/PPy nanocomposite sensor when exposed to 10 consecutive cycles of ammonia (30 ppm). Inset: magnification of one of the sensor-response peaks.

The responses of the WO_3_/PPy show a clear linear relationship with the increase of NH_3_ concentration, as can be seen in Figure 9a. This robust correlation underscores the sensitivity of the sensor and its reliable detection capabilities across varying concentrations of ammonia.

**Figure 10:**
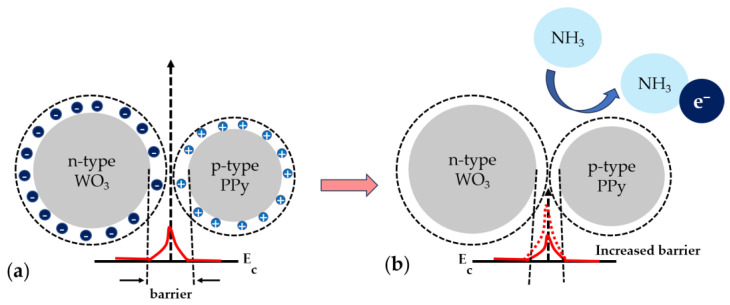
Gas-sensing mechanism for the WO_3_/PPy nanocomposite (**a**) in air and (**b**) when exposed to NH_3_. E_c_ denotes the energy of the conduction band.

Moreover, the repeatability of the WO_3_/PPy gas sensor’s measurements, when exposed to the same concentration of ammonia for a few cycles, has significant importance in gas sensing, as it characterizes the sensor’s capability to consistently yield the same results when subjected to identical conditions repeatedly. This aspect is crucial in assessing the overall reliability of the sensor as well as its suitability for real-world applications. In the present study, the WO_3_/PPy nanocomposite, synthesized using laser-based methods, undergoes thorough evaluation across 10 consecutive cycles, as depicted in Figure 10b. The findings demonstrate remarkably stable fluctuation values when exposed to 30 ppm NH_3_ for successive cycles, with smooth transitions between nitrogen and ammonia gas. Thus, the WO_3_/PPy sensor maintains its performance without significant degradation, contributing to long-term reliability.

The investigations presented in the paper demonstrate that the WO_3_/PPy sensor has better sensing performance, i.e., high response magnitude and repeatability towards 10–50 ppm concentrations of NH_3_ than the sensors based on PPy or WO_3_ alone. Further on, we focus on trying to provide a sensing mechanism responsible for this enhanced functionality. PPy alone is a p-type semiconductor, with holes in the majority of charge carriers. In contrast, WO_3_ is an n-type semiconductor, with electrons as the majority carriers, that does not show an electrical response to NH_3_ at room temperature. Thus, the combination of p-type and n-type behaviour in the WO_3_/PPy nanocomposite gives rise to the formation of p–n heterojunctions. This sensing mechanism has been observed previously in [45,46,47] for polyaniline-SnO_2_ nanocomposites, PPy-ZnO, or PPY-SnO_2_ nanocomposites, etc., where a greater number of p–n heterojunctions correspond to higher sensor efficiency in the composites. Similarly, in our work, if we apply the principle of p–n junction formation, we can say that the electron flow from n-WO_3_ to p–PPy saturates the holes until equilibrium is reached, elevating the Fermi level and equalizing it in both the n and p regions. Additionally, there is a minor electron flow in the reverse direction, i.e., from PPy to WO_3_. This mutual interaction results in charge separation, generating p–n heterojunctions in the PPY–WO_3_ nanocomposite. The p–n heterojunctions formed in the PPY-WO_3_ system could enhance the depletion-barrier height, consequently improving the sensor response, as observed previously, in similar systems [45,46,47,48]. The gas-sensing mechanism of the laser-processed sensing layers is shown in Figure 10. Although we can state that sensing layers fabricated by PLD and MAPLE can be suitable sensing layers in resistor sensors able to detect ammonia gas in the atmosphere at room temperature, additional studies are needed in order to obtain a deep understanding of the enhanced response of the nanocomposites. In addition, our future studies will also focus on the selectivity of the nanocomposite sensors.

Finally, in Table 1, a few performance characteristics of the WO_3_/PPy nanocomposite sensors developed in this work are presented in comparison with reports from the literature.

## 4. Conclusions

Laser-based methods have been applied to synthesize tungsten oxide–polypyrrole nanocomposites. Following the XRD analysis, the WO_3_/PPy nanocomposite was assumed to retain WO_3_ crystalline characteristics. FTIR and Raman spectra of WO_3_/PPy were examined. FTIR bands confirmed a successful combination of PPy and WO_3_. Raman spectra exhibited characteristic peaks of both PPy and WO_3_, indicating no chemical damage from laser processing. Contact-angle measurements were performed to assess surface wettability. WO_3_ and PPy surfaces were hydrophilic, while the WO_3_/PPy nanocomposite surface showed moderate hydrophobicity, which is advantageous for designing ammonia-gas sensors. SEM and AFM images revealed the micro–nano structures of WO_3_, PPy, and WO_3_/PPy layers. WO_3_ exhibited a mesoporous appearance with intricate networks. PPy showed a granular structure, indicating its semicrystalline nature, while the WO_3_/PPy nanocomposite combined the features of both materials. The gas-sensing characteristics were evaluated for PPy, WO_3_, and WO_3_/PPy sensors toward ammonia gas. PPy demonstrated a p-type semiconductor response, while WO3 showed an n-type semiconductor response at 100 ± 1 °C. The WO_3_/PPy nanocomposite exhibited enhanced response and repeatability compared to individual components. The sensor showed a linear relationship with increasing ammonia concentration, emphasizing its sensitivity and reliable detection capabilities across cycles.

In summary, the study demonstrated the successful synthesis and characterization of WO_3_/PPy nanocomposites, showcasing their potential for efficient gas-sensing applications, particularly in detecting ammonia.

## Figures and Tables

**Table 1:** Performance comparison of the WO_3_, PPy, and WO_3_/PPy nanocomposite sensor developed in this work with reports in the literature.

Sensing Material	Fabrication Method	Response (%)	T_res_	T_rec_	Ref.
PPy	Electrochemical deposition	(5–20 ppm)	-	-	[49]
PPY	Chemical polymerization	0.33 (100 ppm)	-	-	[50]
PPy	Chemical polymerization	14 (20 ppm)	20 s	15 min	[51]
PPy multilayered thin films	LBL in situ self assembly	77.75 (50 ppm)	12 s	52 s	[52]
PPy	In situ chemical vapour oxidation	143 (350 ppm)	~60 s	~180 s	[53]
WO_3_ nanoflakes	Hydrothermal	27 (1 ppm)@ 200 °C	>10 min	>10 min	[5]
WO_3_ microspheres	Two-step hydrothermal route	3.32 (100 ppm))@ 350 °C	150 s	200 s	[54]
WO_3_	PLD	2 (50 ppm)@ 300 °C	10–20 s	10–20 s	[8]
WO_3_ *	PLD	0.25 (50 ppm)	80 s	70 s	This work
PPy *	MAPLE	3.5 (50 ppm)	-	15 min	This work
WO_3_/PPy *	PLD–MAPLE	20	50 s	70 s	This work

* better sensing performance.

## Data Availability

The data used to support the findings of this study are available from the corresponding author upon request.

## Supplementary Materials

The following supporting information can be downloaded at: https://www.mdpi.com/article/10.3390/polym16010079/s1, Figure S1: Sketch of the sensor testing setup. MFC = mass-flow controller; SLM = standard litre per minute; SCCM = standard cubic centimetre per minute. Figure S2: (**a**) Raman spectra of WO_3_ sensing layer obtained by PLD. (**b**) Raman spectra of a PPy sensing layer obtained by MAPLE. Table S1: Contact-angle measurements carried out on the active layers obtained by laser methods.
