# Supplementary material for: Polypyrrole–Tungsten Oxide Nanocomposite Fabrication through Laser-Based Techniques for an Ammonia Sensor: Achieving Room Temperature Operation
Source: Polymers (Basel). 2023 Dec 26;16(1):79. doi: 10.3390/polym16010079 (PMC10780584; doi:10.3390/polym16010079)
Supplement: Supplementary file 1 [file polymers-16-00079-s001.zip › polymers-2792504-supplementary.pdf]

polymers

MDPI

Supplementary Information

Polypyrrole–Tungsten Oxide Nanocomposite Fabrication through Laser-Based Techniques for an Ammonia Sensor: Achieving Room Temperature Operation

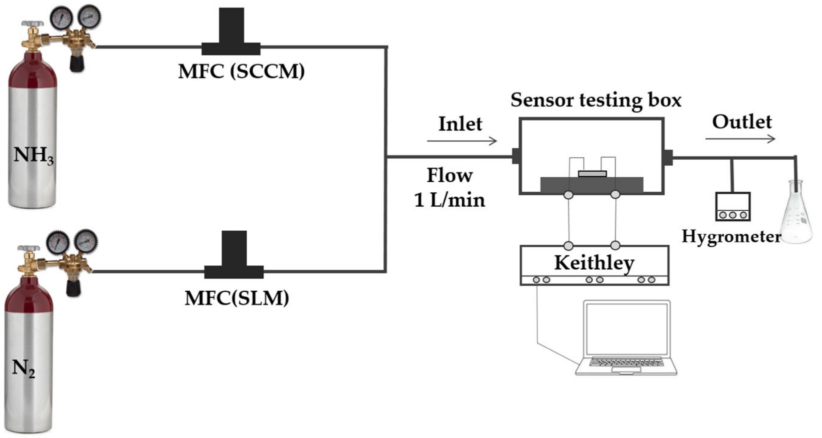

Figure S1. Sketch of the sensor testing setup. MFC = Mass Flow Controller; SLM = Standard Liter per Minute; SCCM = Standard Cubic Centimeter per Minute.

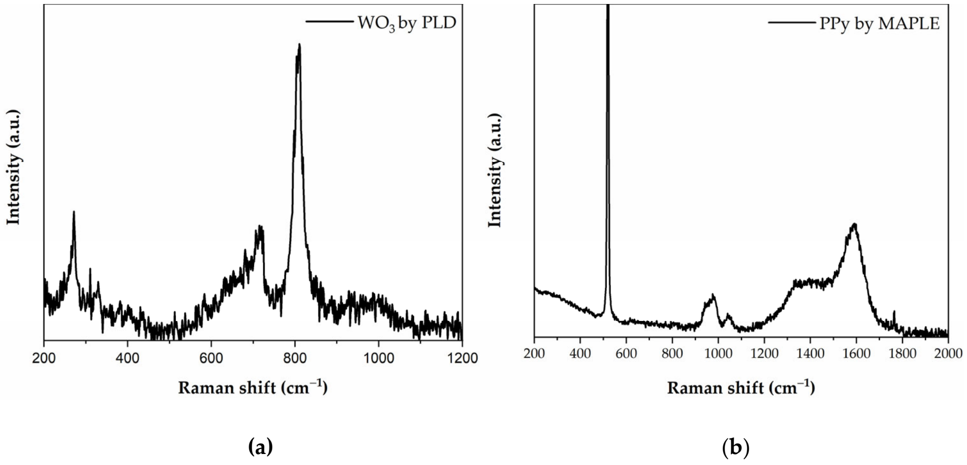

Figure S2. (**a**) Raman spectra of WO_3 sensing layer obtained by PLD. (**b**) Raman spectra of a PPy sensing layer obtained by MAPLE.

Table S1. Contact angle measurements carried out on the active layers obtained by laser-methods.

Layer	m1	m2	m3	m4	m5	Average
WO_3 by PLD	41.38	41.61	41.82	45.26	45.68	**43.15**
PPy by MAPLE	47.42	47.44	47.41	47.55	47.57	**47.45**
WO_3/PPy	63.67	66.12	63.61	63.82	67.42	**64.92**
